# Cigarette smoke alters circRNA expression in human T-cells

**DOI:** 10.12688/f1000research.130430.1

**Published:** 2023-05-30

**Authors:** So Jin Hong, Zhaohao Liao, Kenneth W. Witwer, Ashley E. Russell

**Affiliations:** 1 Department of Molecular and Cellular Biology, Krieger School of Arts & Sciences, Johns Hopkins University, Baltimore, Maryland, USA; 2 Department of Molecular and Comparative Pathobiology, School of Medicine, Johns Hopkins University, Baltimore, Maryland, USA; 3 Department of Neurology, School of Medicine, Johns Hopkins University, Baltimore, Maryland, USA; 4 Richman Family Precision Medical Center of Excellence in Alzheimer's Disease, School of Medicine, Johns Hopkins University, Baltimore, Maryland, USA; 5 Department of Biology, School of Science, Penn State Erie The Behrend College, Erie, Pennsylvania, USA

**Keywords:** Cigarette smoke, T-cells, circRNA, HIV

## Abstract

Circular RNAs (circRNAs), once thought to be a result of splicing errors, have been found to be involved in various molecular processes and the pathology of various diseases, including cancer and neurodegenerative diseases. Additionally, circRNA expression was found to be altered by lifestyle habits, such as smoking cigarettes. Past studies have revealed that the rate of smoking remains high in people living with human immunodeficiency virus (HIV). In this study, we isolated total RNA from uninfected T-cells that have been exposed to cigarette smoke and compared the expression levels of circRNAs to those of T-cells that were not exposed to cigarette smoke. We identified certain circRNAs that were upregulated or downregulated in T-cells when exposed to cigarette smoke. These data indicate that the study of circRNAs is warranted within the context of HIV.

## Introduction

Circular RNAs (circRNAs) are endogenous non-coding RNAs covalently bonded circular structures that are created through back-splicing events ( Yu & Kuo, 2019). CircRNAs are predominantly present in the cytoplasm, but have also been shown to be present in extracellular vesicles (EVs) released from cells ( Gotts *et al.*, 2018; Preußer *et al.*, 2018). Although first thought to be a result of splicing errors, circRNAs have been found to play important roles in various molecular processes, such as functioning as microRNA (miRNA) sponges ( Hansen *et al.*, 2013). miRNAs are small non-coding RNAs that are involved in regulating protein production by binding to complementary sequences in the 3′ untranslated region of messenger RNAs (mRNAs) and preventing their translation into protein ( Jonas & Izaurralde, 2015). By binding miRNAs, circRNAs can alleviate repression of linear mRNA targets and thus influence gene regulation.

In some cancers, including pancreatic and breast cancer, aberrant expression of circRNAs has been observed ( Rong *et al.*, 2021; Wang *et al.*, 2019a, 2019b). Altered expression of circRNAs has also been associated with neurodegenerative diseases such as Alzheimer’s Disease ( Lo *et al.*, 2020). Together, these data suggest that circRNAs are involved in disease pathology and may serve as novel biomarkers for various diseases. Notably, lifestyle habits like cigarette smoking alter circRNA expression as well ( Zeng *et al.*, 2019), indicating that a person’s smoking status may impact epigenetic mechanisms mediated by miRNAs.

Although the rate of smoking in the general population has been on the decline, it remains higher in people living with human immunodeficiency virus (HIV) ( Socio *et al.*, 2020). Individuals living with HIV who smoke tend to have a more serious prognosis or develop other HIV-related diseases such as cancers, pneumonia, and chronic obstructive pulmonary disease (COPD) ( Giles *et al.*, 2018; Hile *et al.*, 2016), the pathophysiology of which may be impacted by epigenetic regulation of miRNAs and circRNAs. In the current study, we sought to investigate the effects of cigarette smoke on circRNA expression profiles in a model of uninfected human T-cells (H9 cells) to understand how cigarette smoke impacts circRNA expression in this cell type, a common target of HIV ( Walker & McMichael, 2012). These data will provide a foundation for investigating circRNA expression in HIV-infected H9 cells that have been exposed to cigarette smoke to understand how these two variables intersect.

## Methods

### Preparation of 100% cigarette smoke extract media (CSEM)

Cigarette smoke extract (CSE) was made using an apparatus and techniques as previously described by our lab ( Bernstein *et al.*, 2019). Briefly, 25 mL of R10 media was aliquoted into a 50 mL conical tube and sealed with parafilm. A 5 mL serological pipette, cut at the 3.5 mL mark, was inserted through the parafilm seal. This apparatus was connected to one end of a Tygon tubing (Cole-Parmer, Cat. No. 06509-17), which was inserted into a Cole-Parmer Masterflex L/S peristaltic pump at speed setting of 32. A 1 mL pipette tip was tightly inserted into the other end of the Tygon tubing. Next, one Spectrum research-grade cigarette ( Richter *et al.*, 2016) (filter intact) was lit with a Bunsen burner and tightly inserted into the wide end of the 1 mL pipette tip. The cigarette was then smoked continuously by the peristaltic pump into the cell culture medium. The resulting product was considered 100% CSE.

### H9 cell cigarette smoke exposure

Approximately 400,000 cells/mL of H9 cells were passaged into six T75 flasks, three of which were filled with 25 mL of R10 media with 0% CSEM and the other three with 25 mL of 50% CSEM. After 24 hours, the percentage of viable cells was measured ( Table 1) using a Muse cell counter and Muse Count & Viability Reagent (Luminex, Cat. No. MCH600103).

**Table 1.** Percentage of viable H9 cells. Percentages of cell viability and density were measured and calculated after 24 hours for six flasks of H9 cells: three with 0% CSEM for 24 hours and three with 50% CSEM. CSEM, cigarette smoke extract media.

% CSEM	Viable cells/mL	% Viability
0% - flask #1	1,195,679	98.1
0% - flask #2	1,196,918	97.1
0% - flask #3	1,248,749	96.7
50% - flask #1	723,372.7	85.9
50% - flask #2	632,603.8	86.1
50% - flask #3	715,714.1	87.9

### Total RNA and circRNA isolation

The Invitrogen mirVana™ miRNA Isolation Kit (ThermoFisher Scientific, Cat. No. AM1560) was used as directed to isolate total cellular RNA. Approximately 10 ^5^ cells from each flask were lysed with 600 μL lysis buffer. Then, 750 μL 100% ethanol was added and centrifuged at 10,000 × g for 15 seconds. The filters containing RNA were washed, and total RNA was eluted with 100 μL pre-heated nuclease-free water, centrifuging at maximum speed for 25 seconds. RNA quality was checked using an Agilent Fragment Analyzer. Next, 5,000 ng of RNA from each sample was shipped to Arraystar for hybridization with the Human Circular RNA Array (Cat. No. AS-S-CR-H-V2.0).

### Human circRNA array, data processing, and analysis

Total RNAs from each sample were quantified using the NanoDrop ND-1000 and the integrity of the samples were evaluated through electrophoresis on a denaturing agarose gel. Total RNAs were then digested with RNase R (Epicentre, Inc.) to degrade linear RNAs and enrich circRNAs. A random priming method was used to amplify and transcribe the enriched circRNAs into fluorescent complementary RNAs (cRNAs) (Arraystar Super RNA Labeling Kit; Arraystar), which were then purified by RNeasy Mini Kit (Qiagen). Nanodrop ND-1000 was used to measure the concentration and specific activity of the labeled cRNAs (pmol Cy3/μg cRNA). A total of 1 μg of each labeled cRNA was fragmented by adding 5 μl of 10× Blocking Agent and 1 μl of 25× Fragmentation Buffer. The mixture was then incubated at 60°C for 30 minutes, then 2× Hybridization buffer was added to dilute the labeled cRNA. Then, 50 μl of the hybridization solution was dispensed into the gasket slide and assembled to the circRNA expression microarray slides, which were washed, fixed, and scanned with the Agilent Scanner G2505C.

Agilent Feature Extraction Software (version 11.0.1.1) (RRID:SCR_014963) was used to extract raw data from the scanned images. Quantile normalization of the raw data and subsequent data processing were conducted using the R software Limma package (RRID:SCR_010943). Low intensity filtering was then performed to retain circRNAs that at least one out of six samples were flagged as “present” or “marginal”. The quality control flags of “present”, “marginal”, or “absent” were determined based on various features, including positive and significant signal, saturation, population outlier, above background, and uniformity of the background. The fold change between the cigarette exposed samples and control samples were calculated and the statistical significance of the difference was estimated using an unpaired t-test. circRNAs having fold changes greater than or equal to 1.5 and p-values less than or equal to 0.05 were selected as significantly differentially expressed, using Microsoft Excel (RRID:SCR_016137). The false detection rate for each differentially expressed circRNAs were calculated using Benjamini-Hochberg procedure. These data, while suggestive, do not support robust statistical analysis secondary to the number of repeats and the large number of RNAs.

## Dataset

The Human Circular RNA Array revealed 234 circRNAs that were 1.5-fold upregulated (Up Regulated circRNAs) and 42 circRNAs that were 1.5-fold downregulated (Down Regulated circRNAs) in H9 cells cultured in 50% CSEM relative to H9 cells grown in 0% CSEM ( Hong *et al.*, 2023).

## Data Availability

Gene Expression Omnibus: Cigarette Smoke Alters CircRNA Expression in Human T-Cells. GSE228979; https://identifiers.org/geo/GSE228979 ( Hong *et al.*, 2023). This series contains the following underlying data:
- Datasheet 1 (1.5 Fold Up Regulated circRNAs. Datasheet 1 contains raw and normalized fluorescence intensities from the ArrayStar circRNA Microarray, for 234 circRNAs that were significantly upregulated in H9 cells after cigarette smoke exposure. The circRNA ID was obtained by inputting the transcript and sequence information into circBase or other literatures). - Datasheet 2 (1.5 Fold Down Regulated CircRNAs. Datasheet 2 contains raw and normalized fluorescence intensities from the ArrayStar circRNA Microarray, for 42 circRNAs that were significantly downregulated in H9 cells after cigarette smoke exposure. The circRNA ID was obtained by inputting the transcript and sequence information into circBase or PMIDs).
