# Supplementary material for: Cigarette smoke alters circRNA expression in human T-cells
Source: F1000Res. 2025 Apr 9;12:566. Originally published 2023 May 30. [Version 3] doi: 10.12688/f1000research.130430.3 (PMC11831728; doi:10.12688/f1000research.130430.3)
Supplement: Peer review history

# Reviewer report: Reviewer response for version 2

**DOI:** 10.5256/f1000research.167219.r359113

**Published:** 2025-02-13

**Authors:** Meity Ardiana

**Affiliations:** 1 Universitas Airlangga, Surabaya, Indonesia

You provided a clear and well-researched background on circRNAs, their formation, and their functional significance. The explanation of circRNAs as miRNA sponges is well-supported with references, also, the introduction connects circRNAs to major diseases like cancer and neurodegenerative disorders which increases the study's significance and valuable. However, there are some suggestions that I think you could consider adding. The connection between smoking, HIV, and disease progression is mentioned, but explaining why Tcells are the main focus would make the rationale stronger.

 Consider briefly explaining:

 ˗ Why H9 cells specifically (e.g., their relevance as a model for HIV studies)?

 ˗ If circRNA research in HIV-T cells is novel, highlight this gap The discussion on HIV-related diseases could be slightly expanded.

 For instance: ˗

 What is the known impact of smoking on immune cells in the context of HIV?

 ˗ Are there any prior studies linking smoking to circRNA expression in immune cells?

 You mentioned that smoking rates are higher among people with HIV, but it would help to briefly explain why. Is it due to behavioral, socioeconomic, or biological factors? Regarding the study's aim, if the ultimate goal is to study HIV-infected cells, briefly mention how these findings might help in future experiments. You've already mentioned that you're trying to understand how the variables intersect, which is a good purpose base, but in what way do you hope that this data help other researchers (probably for those who are studying similar case)?

Are sufficient details of methods and materials provided to allow replication by others?

Partly

Is the rationale for creating the dataset(s) clearly described?

Yes

Are the datasets clearly presented in a useable and accessible format?

Yes

Are the protocols appropriate and is the work technically sound?

Partly

Reviewer Expertise:

I am quite knowledgeable in stem cell

I confirm that I have read this submission and believe that I have an appropriate level of expertise to confirm that it is of an acceptable scientific standard, however I have significant reservations, as outlined above.

# Response

**Published:** 2025-04-01

**Authors:** So Jin Hong

**Affiliations:** Johns Hopkins Medicine, USA

Dear Dr. Meity Ardiana,

 Thank you for giving us the opportunity to submit a second revised draft of our manuscript. We truly appreciate the time and effort that you have dedicated to providing your valuable feedback. We are grateful for your insightful comments on our paper. We have been able to incorporate changes to reflect most of the suggestions provided. We have highlighted the changes within the manuscript.

 Here is a point-by-point response to your comments and concerns.

**Comment 1: **Why H9 cells specifically (e.g. their relevance as a model for HIV studies)?

**Response:** This is a great point. We have included in the Methods section “H9 cell cigarette smoke exposure” our rationale for using H9 cells: “H9 cells were used for this experiment specifically for their susceptibility to HIV-1 infections”. These cells are often utilized in immunology research and were used for this experiment specifically for their susceptibility to HIV-1 infections. 

**Comment 2:** If circRNA research in HIV-T cells is novel, highlight this gap. The discussion on HIV-related diseases could be slightly expanded. For instance: What is the known impact of smoking on immune cells in the context of HIV? Are there any prior studies linking smoking to circRNA expression in immune cells?

**Response: **Thank you for pointing this out. At the time our experiments were conducted, the relationship between cigarette smoke and circRNA expression was not well known. Since writing of this manuscript, a manuscript written by Shen et al., titled Circular RNA Expression of Peripheral Blood Mononuclear Cells Associated with Risk of Acute Exacerbation in Smoking Chronic Obstructive Pulmonary Disease found a strong correlation between smoking and expression levels of unique circRNAs (hsa_circ_0049875 and hsa_circ_0042590) in COPD peripheral blood mononuclear cells, and the frequency of acute exacerbations was shown to be substantially linked with these circRNA expressions. These recent findings further emphasize the importance of circRNA research in the setting of cigarette smoke exposure and diseases affecting immune cells, such as HIV. This information was added towards the end of the Introduction section.

**Comment 3:** You mentioned that smoking rates are higher among people with HIV, but it would help to briefly explain why. Is it due to behavioral, socioeconomic, or biological factors? 

**Response: **We agree with your suggestion. Upon looking into the literature, the persistently high rate of smoking in people living with HIV has been found to be due to combinations of complex psychosocial characteristics and unhealthy behavioral factors, including but not limited to: lower education and economic levels, unemployment, depression, and drug usage. Information has been added to the third paragraph of the Introduction section. 

**Comment 4: **Regarding the study’s aim, if the ultimate goal is to study HIV-infected cells, briefly mention how these findings might help in future experiments. You’ve already mentioned that you’re trying to understand how the variables intersect, which is a good purpose base, but in what way do you hope that this data help other researchers (probably for those who are studying similar case)?

**Response:** Thank you for pointing this out. We believe the data published in the manuscript could be utilized as a foundation for further exploration of circRNAs affected by cigarette smoke in HIV infected T-cells. This information has been added at the end of the third paragraph of the Introduction section.

 We look forward to hearing from you regarding our edits and to respond to any further questions and comments you may have. 

 Sincerely,

 So Jin (Diane) Hong

# Reviewer report: Reviewer response for version 2

**DOI:** 10.5256/f1000research.167219.r291433

**Published:** 2024-07-29

**Authors:** Zahraa Rahal

**Affiliations:** 1 University of Texas MD Anderson Cancer, Houston, USA

No additional comments.

Are sufficient details of methods and materials provided to allow replication by others?

Partly

Is the rationale for creating the dataset(s) clearly described?

Partly

Are the datasets clearly presented in a useable and accessible format?

Yes

Are the protocols appropriate and is the work technically sound?

Partly

Reviewer Expertise:

I study smoking-induced (cigarette and waterpipe) lung cancer.

I confirm that I have read this submission and believe that I have an appropriate level of expertise to confirm that it is of an acceptable scientific standard.

# Reviewer report: Reviewer response for version 1

**DOI:** 10.5256/f1000research.143189.r255365

**Published:** 2024-04-01

**Authors:** Zahraa Rahal

**Affiliations:** 1 University of Texas MD Anderson Cancer, Houston, USA

The article investigates the impact of cigarette smoke on circRNA expression in uninfected human T-cells (H9 cells), motivated by the high rate of smoking in people living with human immunodeficiency virus (HIV). It explores circRNAs' role in molecular processes and their potential alteration due to lifestyle habits, like smoking, which may affect the pathology of various diseases. The study employed cigarette smoke extract media to expose H9 cells to cigarette smoke and analyzed changes in circRNA expression using RNA isolation, circRNA enrichment, and microarray analysis.

 While the connection between circRNA expression and lifestyle factors like smoking is intriguing, the rationale for focusing specifically on uninfected T-cells and its direct relevance to HIV-positive individuals remains underexplained. The leap from smoking's impact on circRNA in a general context to its implications for HIV-specific pathology could be more robustly justified with existing literature or preliminary data. To strengthen the rationale, the authors should provide a more detailed synthesis of existing literature or preliminary data that directly connects circRNA alterations due to smoking with HIV pathology. Additionally, clarifying why uninfected T-cells serve as an appropriate model for this investigation would help solidify the study's foundation.

 The methods are detailed, but there's a lack of information regarding the validation of the experimental setup. For example, the efficiency and reproducibility of the cigarette smoke extraction method and its physiological relevance to real-world smoking exposure might raise questions. It might be useful to include a validation section for the CSE method, comparing it to physiological conditions or other established methods.

Are sufficient details of methods and materials provided to allow replication by others?

Partly

Is the rationale for creating the dataset(s) clearly described?

Partly

Are the datasets clearly presented in a useable and accessible format?

Yes

Are the protocols appropriate and is the work technically sound?

Partly

Reviewer Expertise:

I study smoking-induced (cigarette and waterpipe) lung cancer.

I confirm that I have read this submission and believe that I have an appropriate level of expertise to confirm that it is of an acceptable scientific standard, however I have significant reservations, as outlined above.

# Response

**Published:** 2024-05-28

**Authors:** So Jin Hong

**Affiliations:** Johns Hopkins Medicine, USA

Dear Dr. Zahraa Rahal,

 Thank you for giving us the opportunity to submit a revised draft of our manuscript. We appreciate the time and effort that you have dedicated to providing your valuable feedback. We are grateful for your insightful comments on our paper. We have been able to incorporate changes to reflect most of the suggestions provided. We have highlighted the changes within the manuscript.

 Here is a point-by-point response to your comments and concerns.

- **Comment 1**: While the connection between circRNA expression and lifestyle factors like smoking is intriguing, the rationale for focusing specifically on uninfected T-cells and its direct relevance to HIV-positive individuals remains underexplained. The leap from smoking's impact on circRNA in a general context to its implications for HIV-specific pathology could be more robustly justified with existing literature or preliminary data. To strengthen the rationale, the authors should provide a more detailed synthesis of existing literature or preliminary data that directly connects circRNA alterations due to smoking with HIV pathology. Additionally, clarifying why uninfected T-cells serve as an appropriate model for this investigation would help solidify the study's foundation.
**Response:** Thank you for pointing this out. T-cells served as an appropriate model for this study, given their susceptibility to HIV infections. Our study using the uninfected T-cells would serve as a baseline for what happens in these cells when exposed to cigarette smoke. The next step of this experiment would be to replicate the study with HIV infected T-cells to study the effects of cigarette smoke in these cells. This information was added to the introduction and the “H9 cell cigarette smoke exposure” methods section.
- **Comment 2**: The methods are detailed, but there's a lack of information regarding the validation of the experimental setup. For example, the efficiency and reproducibility of the cigarette smoke extraction method and its physiological relevance to real-world smoking exposure might raise questions. It might be useful to include a validation section for the CSE method, comparing it to physiological conditions or other established methods.
**Response:** We agree with your suggestion. However, it is difficult to validate the composition of the CSE, therefore we have followed the exact same paradigm across all experiments and our methodology aligns very closely with other published studies in the field. References have been added to our manuscript.

We look forward to hearing from you regarding our edits and to respond to any further questions and comments you may have.

 Sincerely,

 So Jin Hong

# Reviewer report: Reviewer response for version 1

**DOI:** 10.5256/f1000research.143189.r197877

**Published:** 2023-09-18

**Authors:** Thivanka Muthumalage

**Affiliations:** 1 Purdue University, West Lafayette, Indiana, USA

The authors have attempted to show the involvement of circRNA upon exposure to cigarette smoke in HIV infection. While this is important to study, the experimental design lacks information to reproduce the data:

- Cigarette smoke extracts need meticulous standardization as they quickly change their chemical composition. The authors need to elaborate, and specify the brand of the cigarette and how the extract was standardized each time during preparation. 
- Cell culture treatments need more details, such as if they were serum-deprived (if not, what is the serum concentration?) and what passage was used. Please explain the rationale for using a cancer cell line. 
- Please explain the key altered circRNAs and how they are relevant in HIV progression. 
- There are no results and discussion sections; the implications are hard to find.

Overall, while the circRNA alterations may be useful, the authors have not provided sufficient data or discussion for any translational implications. Major revisions are required before indexing.

Are sufficient details of methods and materials provided to allow replication by others?

No

Is the rationale for creating the dataset(s) clearly described?

Partly

Are the datasets clearly presented in a useable and accessible format?

Partly

Are the protocols appropriate and is the work technically sound?

Partly

Reviewer Expertise:

Inhalation toxicology and lung biology

I confirm that I have read this submission and believe that I have an appropriate level of expertise to confirm that it is of an acceptable scientific standard, however I have significant reservations, as outlined above.

# Response

**Published:** 2024-05-28

**Authors:** So Jin Hong

**Affiliations:** Johns Hopkins Medicine, USA

Dear Dr. Thivanka Muthumalage,

 Thank you for giving us the opportunity to submit a revised draft of our manuscript. We appreciate the time and effort that you have dedicated to providing your valuable feedback. We are grateful for your insightful comments on our paper. We have been able to incorporate changes to reflect most of the suggestions provided. We have highlighted the changes within the manuscript.

 Here is a point-by-point response to your comments and concerns.

- **Comment 1**: Cigarette smoke extracts need meticulous standardization as they quickly change their chemical composition. The authors need to elaborate, and specify the brand of the cigarette and how the extract was standardized each time during preparation.

**Response**: Thank you for pointing this out. Spectrum research grade cigarettes that are tightly regulated by the FDA, and distributed by the NIDA, were used following the general techniques utilized by similar studies to help standardize the preparation step. This information has been added to page 3, under the “Preparation of 100% CSEM” methods section.

- **Comment 2**: Cell culture treatments need more details, such as if they were serum-deprived (if not, what is the serum concentration?) and what passage was used. Please explain the rationale for using a cancer cell line.

**Response**: We agree with your suggestion and have incorporated more information regarding the cell culture treatment, passages used, and the rationale behind using the H9 cell lines throughout the manuscript.

- **Comment 3**: Please explain the key altered circRNAs and how they are relevant in HIV progression. 

**Response**: Thank you for your suggestion. It would have been interesting to include this information. However, this is beyond the scope of the current format for this publication as a Data Note, which does not require a Results and Discussion section per F1000Research article guidelines.

- **Comment 4**: There are no results and discussion sections; the implications are hard to find.

**Response**: Thank you for this suggestion. However, our paper was published as a Date Note, which does not require a Results and Discussion section as per F1000Research article guidelines.

 We look forward to hearing from you regarding our edits and to respond to any further questions and comments you may have.

 Sincerely,

 So Jin Hong
